# Supplementary material for: Synthesis, Characterization, and In Vivo Anti-Cancer Activity of New Metal Complexes Derived from Isatin-N(4)antipyrinethiosemicarbazone Ligand Against Ehrlich Ascites Carcinoma Cells
Source: Molecules. 2019 Sep 11;24(18):3313. doi: 10.3390/molecules24183313 (PMC6766913; doi:10.3390/molecules24183313)
Supplement: Supplementary file 1 [file molecules-24-03313-s001.pdf]

SUPPLEMENTARY MATERIALS

Synthesis, Characterization, and In Vivo Anti-Cancer Activity of New Metal Complexes Derived from Isatin-*N*(4)antipyrinethiosemicarbazone Ligand Against Ehrlich Ascites Carcinoma Cells

Fathy El-Saied [1], Bishoy El-Aarag [2,3,*], Tarek Salem [4], Ghada Said [1], Shaden A. M. Khalifa [5,6] and Hesham R. El-Seedi [1,7,8,9,*]

[1] Department of Chemistry, Faculty of Science, Menoufia University, Shebin El-Koom 32512, Egypt; elsayedfathy139@yahoo.com (F.E.-S.); ghadasaid_2007@yahoo.com (G.S.)
[2] Biochemistry Division, Chemistry Department, Faculty of Science, Menoufia University, Shebin El-Koom 32512, Egypt
[3] Division of Chemistry and Biotechnology, Graduate School of Natural Science and Technology, Okayama University, Okayama 7008530, Japan
[4] Department of Molecular Biology, Genetic Engineering & Biotechnology Institute, University of Sadat City, Sadat City 32958, Egypt; salem_tarek@yahoo.com
[5] Department of Molecular Biosciences, The Wenner-Gren Institute, Stockholm University, SE 106 91 Stockholm, Sweden; shaden.khalifa@su.se
[6] Department of Experimental Cancer Medicine (ECM); Novum, 14157 Huddinge, Stockholm, Sweden
[7] Pharmacognosy Group, Department of Medicinal Chemistry, Uppsala University, Biomedical Centre, Box 574, SE-751 23 Uppsala, Sweden
[8] International Research Center for Food Nutrition and Safety, Jiangsu University, Zhenjiang 212013, China
[9] Al-Rayan Research and Innovation Center, Al-Rayan Colleges, Medina, 42541, Saudi Arabia
* Correspondence: bishoy.yousef@gmail.com (B.E.-A.); hesham.el-seedi@ilk.uu.se (H.R.E.-S.); Tel.: +20-1271732703 (B.E.-A.); +46-700434343 (H.R.E.-S.)

^{1}H-NMR spectra

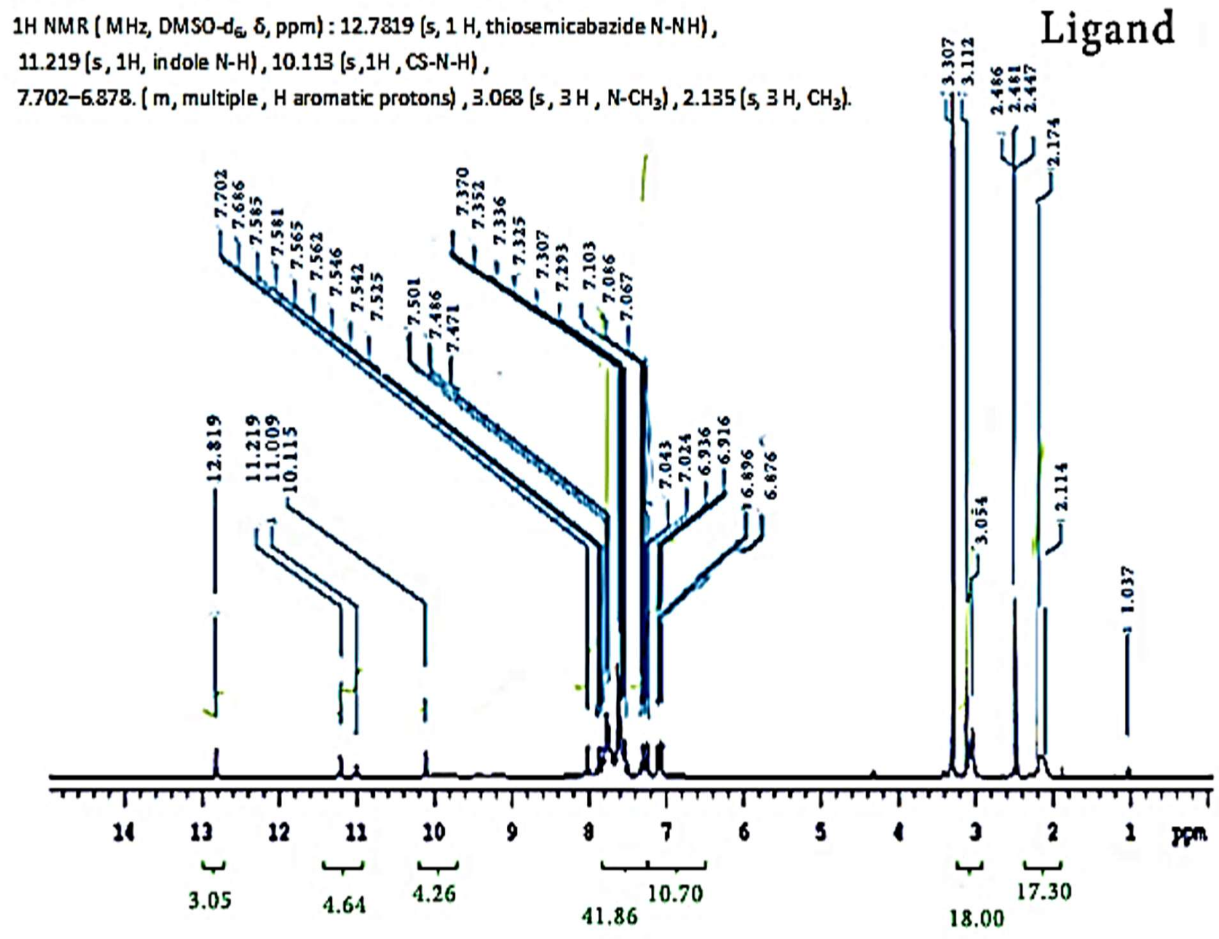

^{1}H-NMR spectra of the ligand **1**.

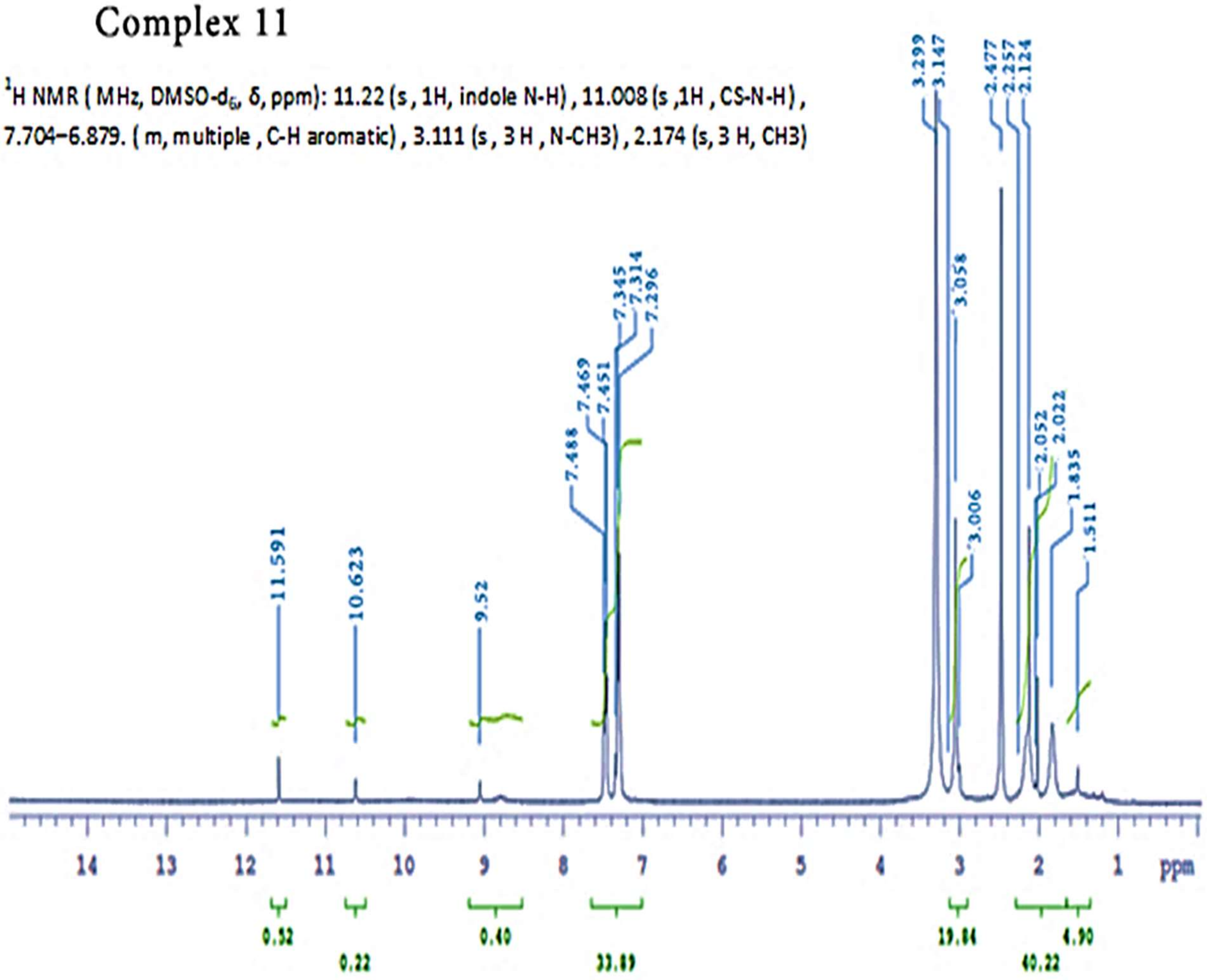

^{1}H-NMR spectra of complex **11**.

Mass spectra

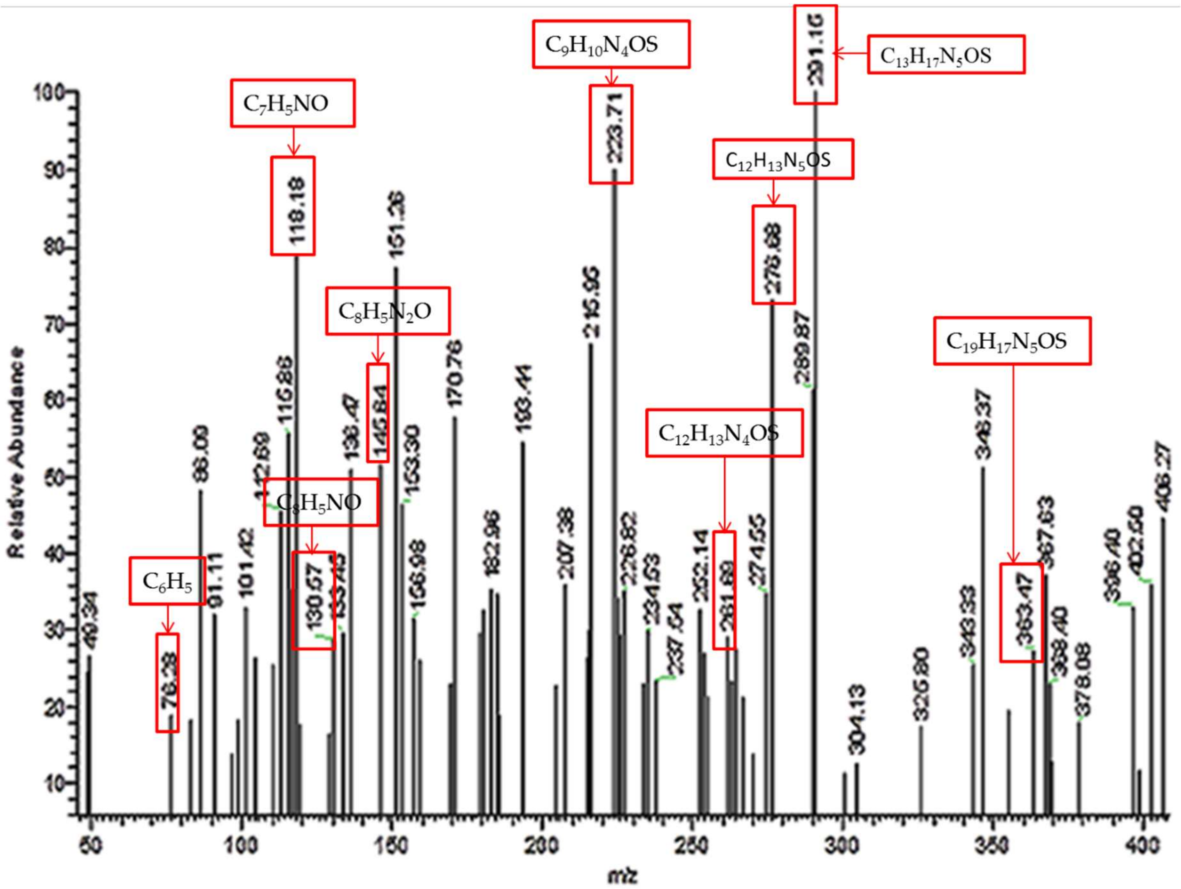

Mass spectra of ligand **1**

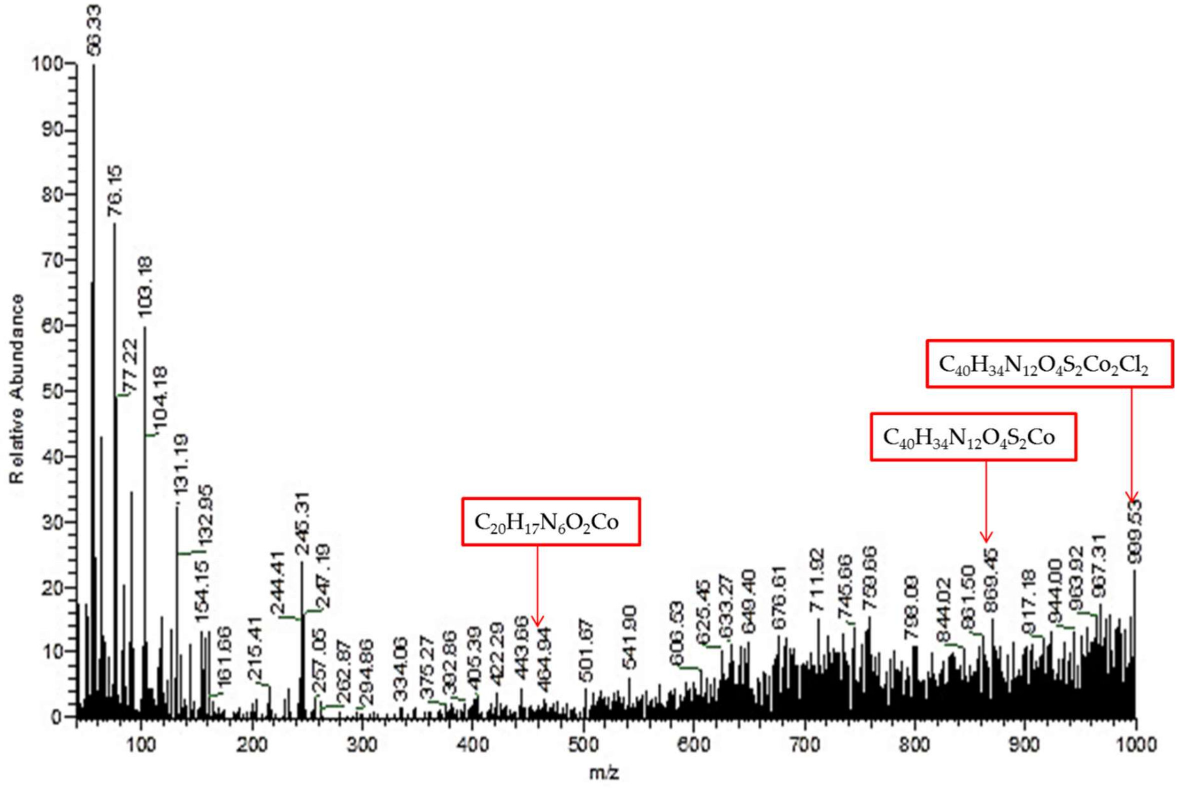

Mass spectra of complex **7**

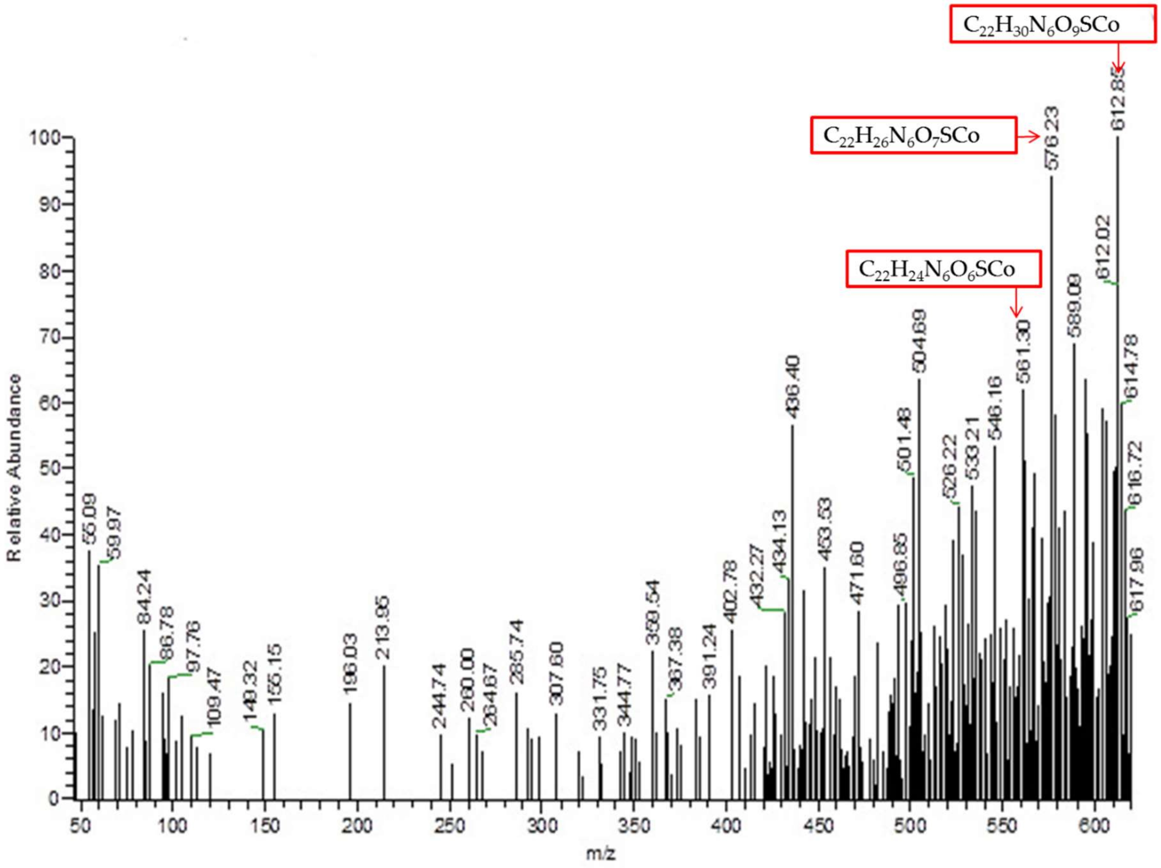

Mass spectra of complex **8**

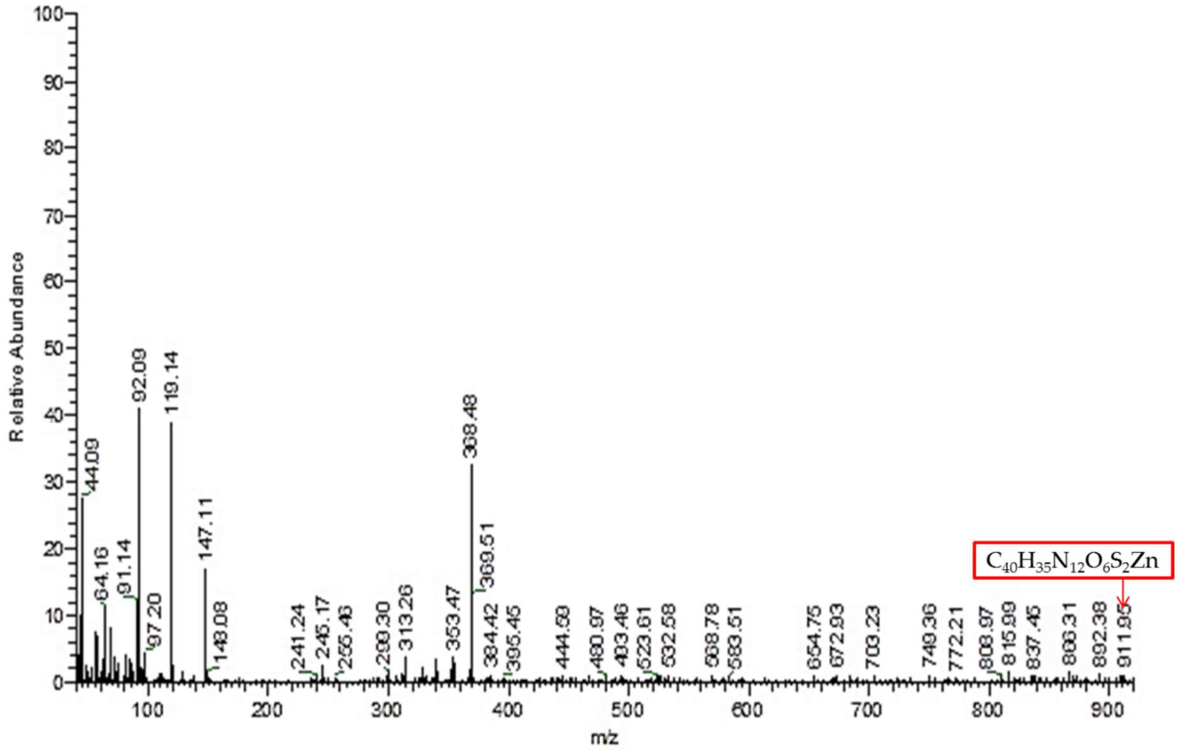

Mass spectra of complex **11**

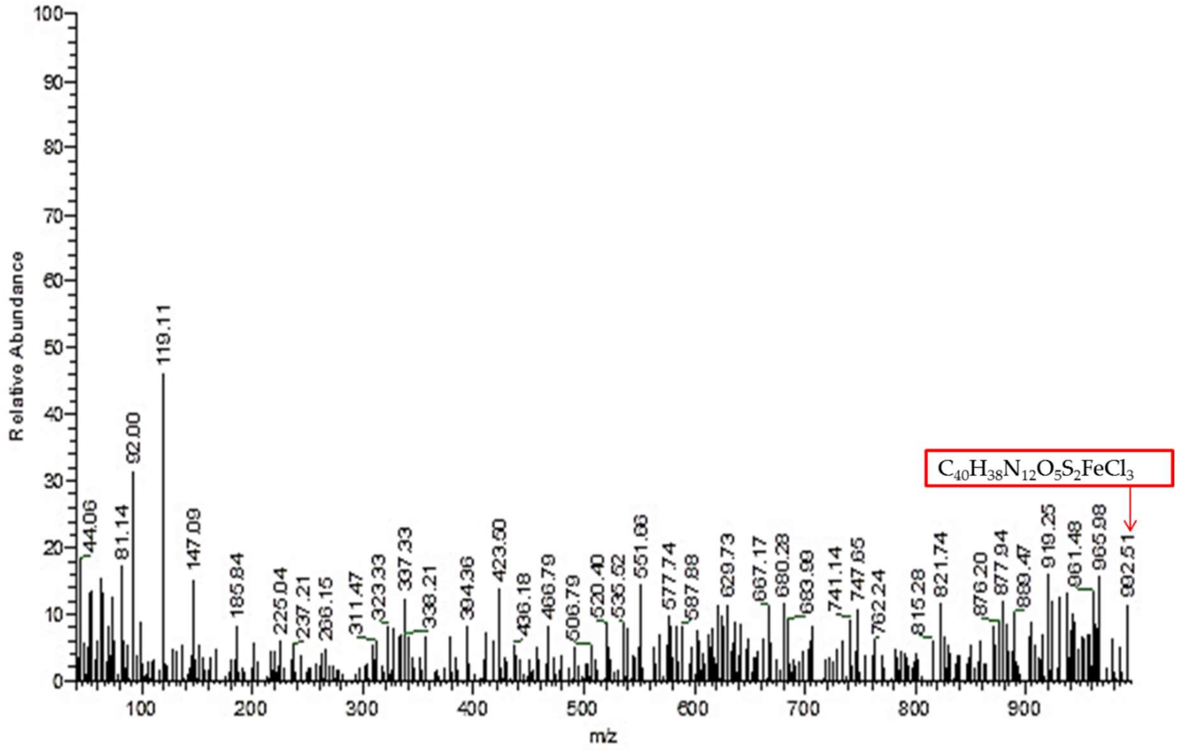

Mass spectra of complex **12**

IR spectra

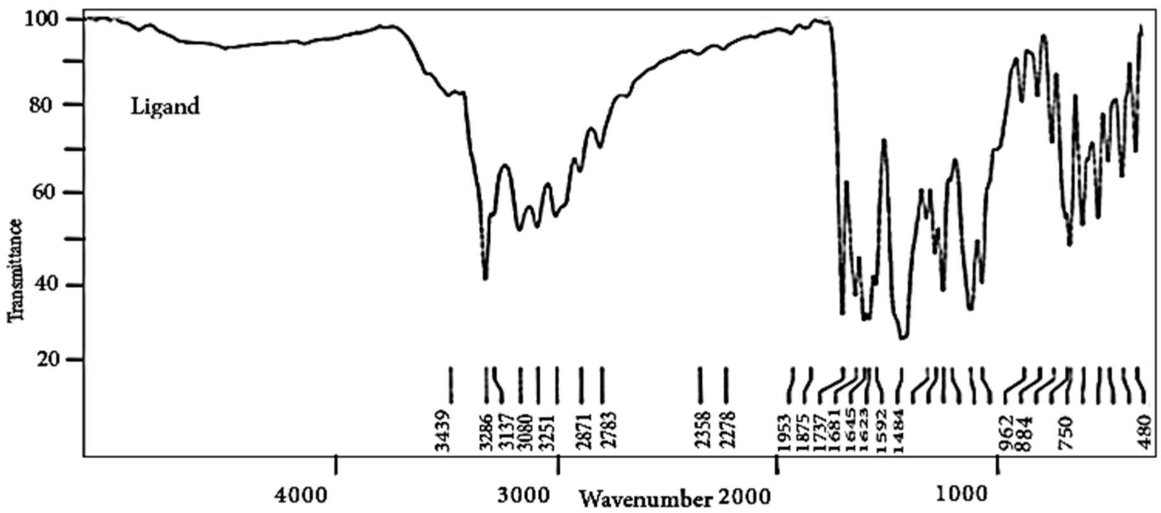

IR spectra of ligand

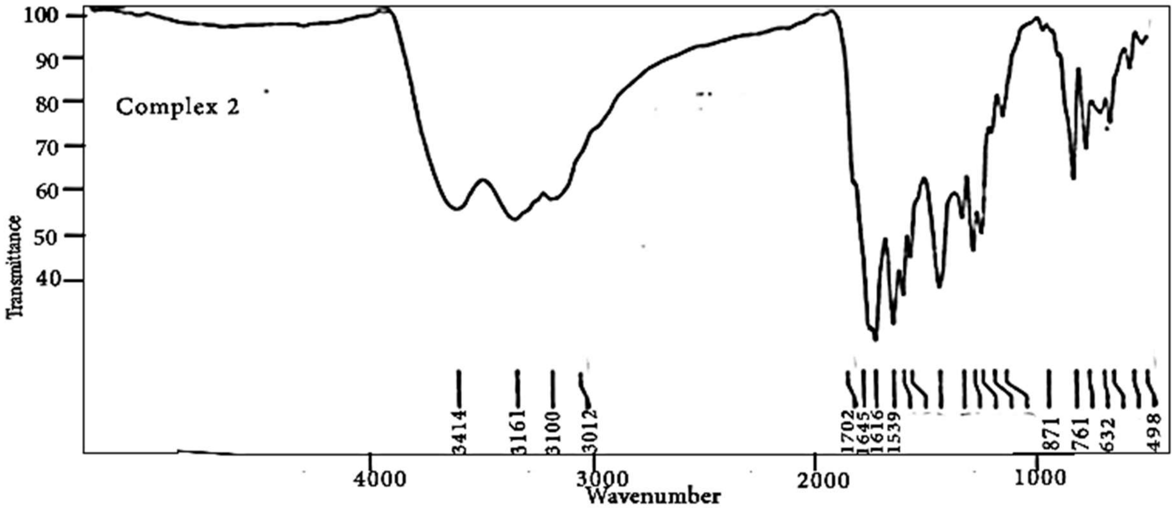

IR spectra of complex **2**

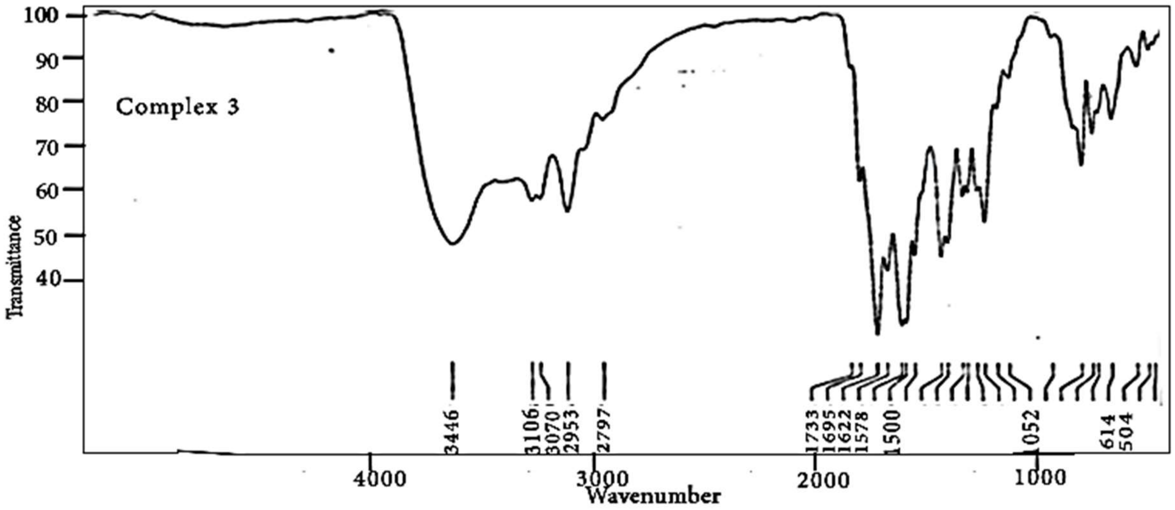

IR spectra of complex **3**

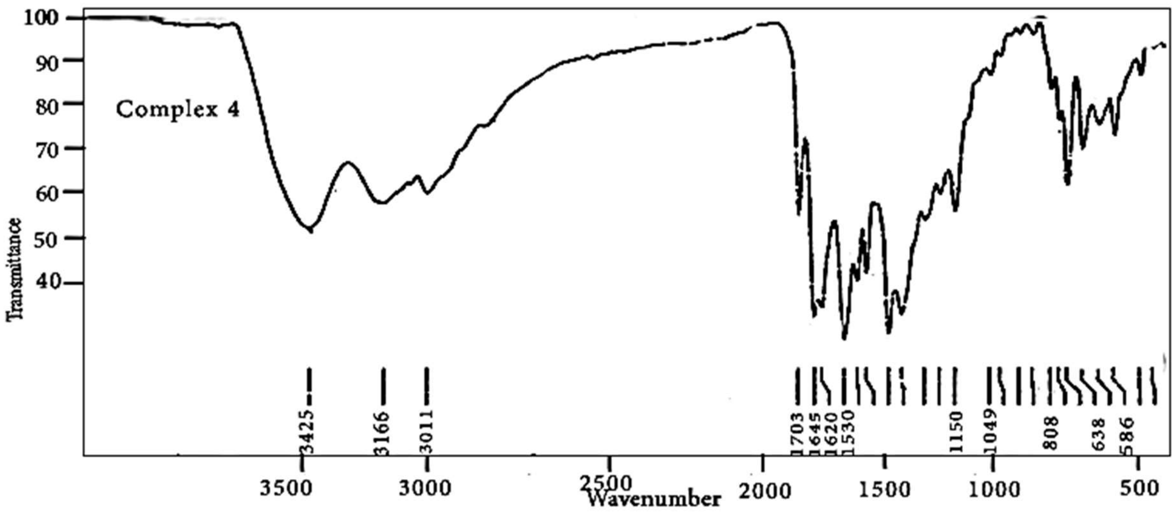

IR spectra of complex **4**

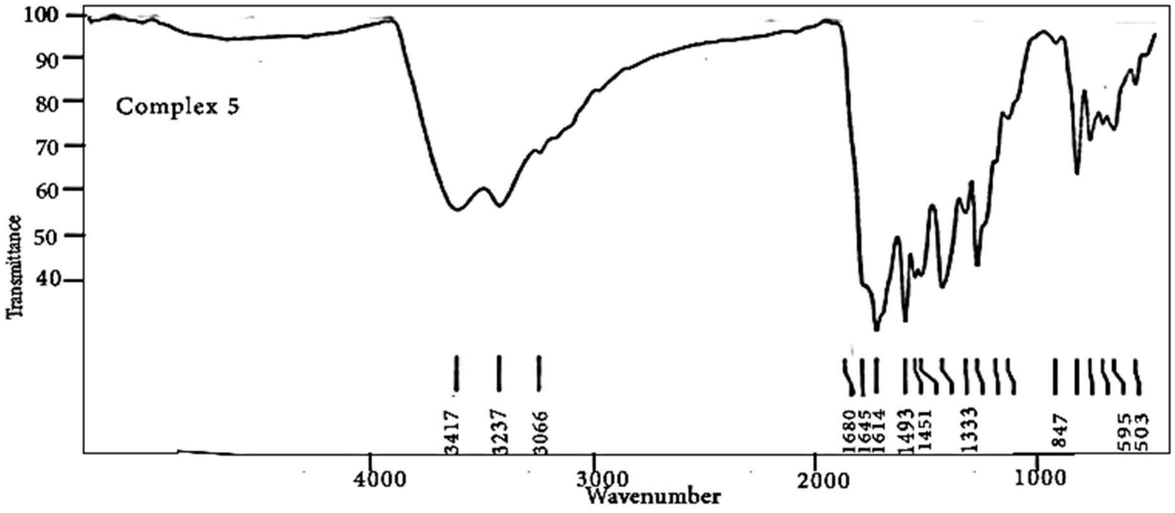

IR spectra of complex **5**

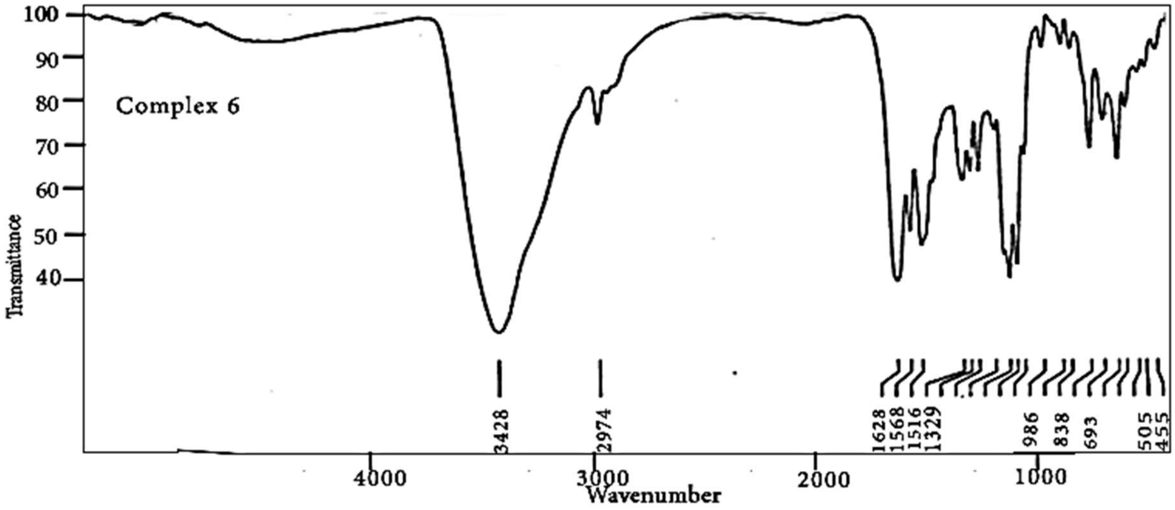

IR spectra of complex **6**

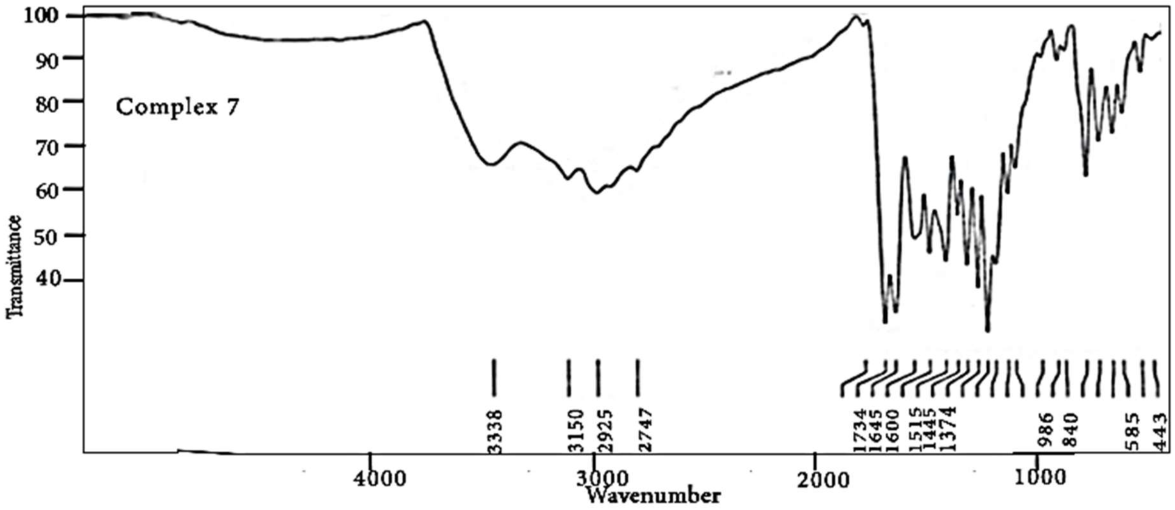

IR spectra of complex **7**

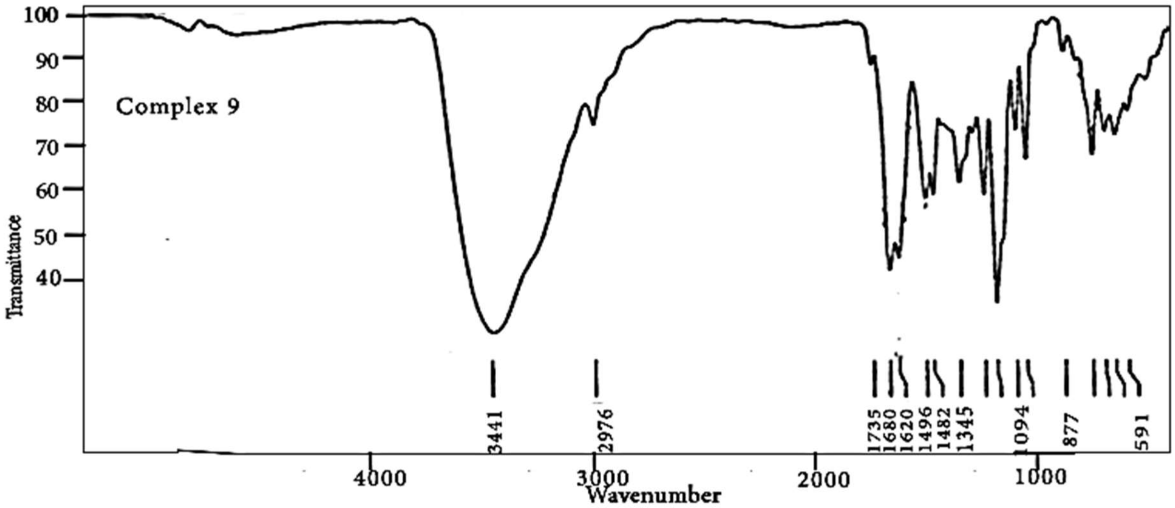

IR spectra of complex **9**

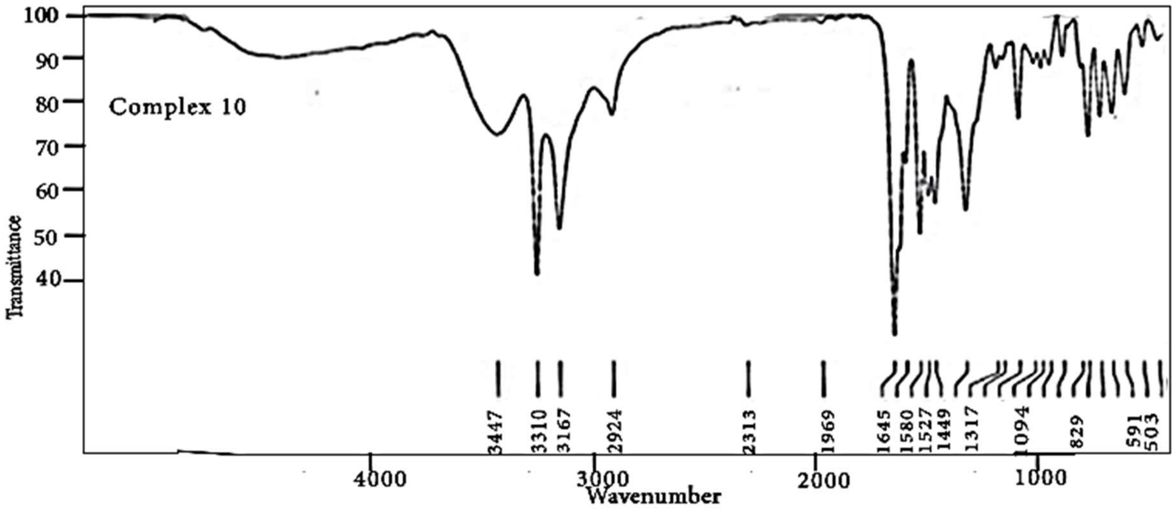

IR spectra of complex **10**

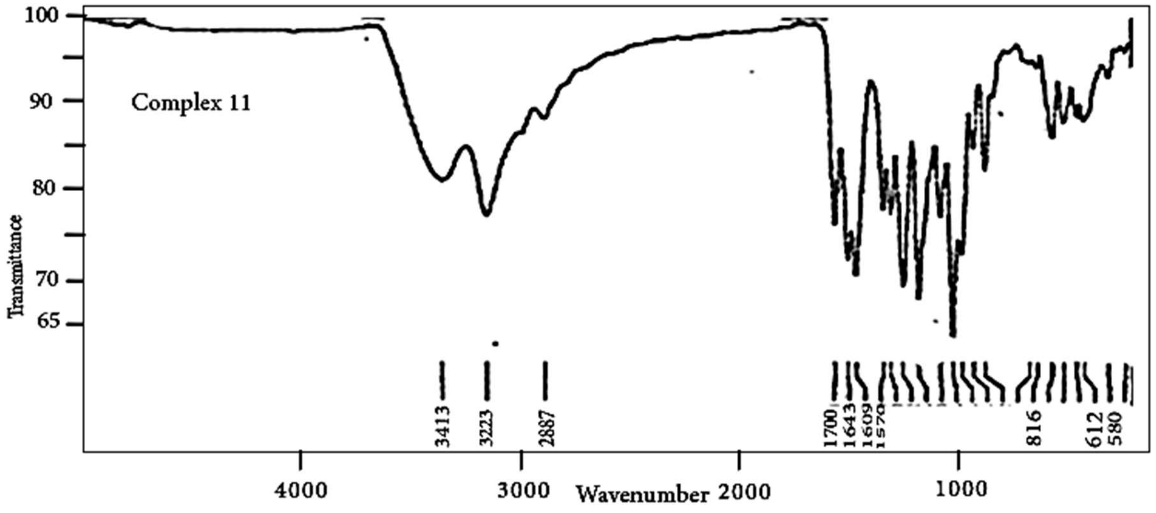

IR spectra of complex **11**

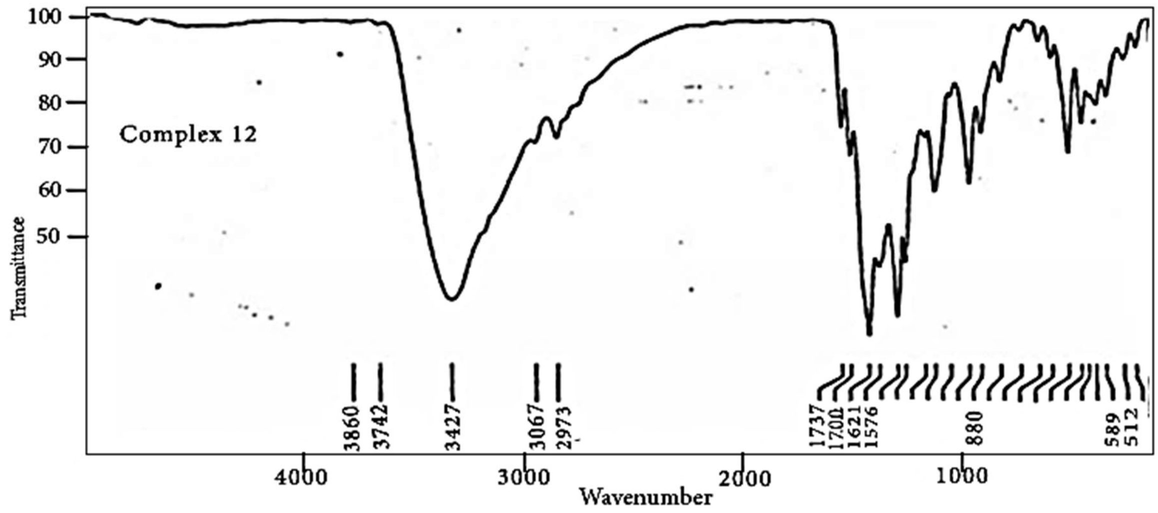

IR spectra of complex **12**

EPR spectra

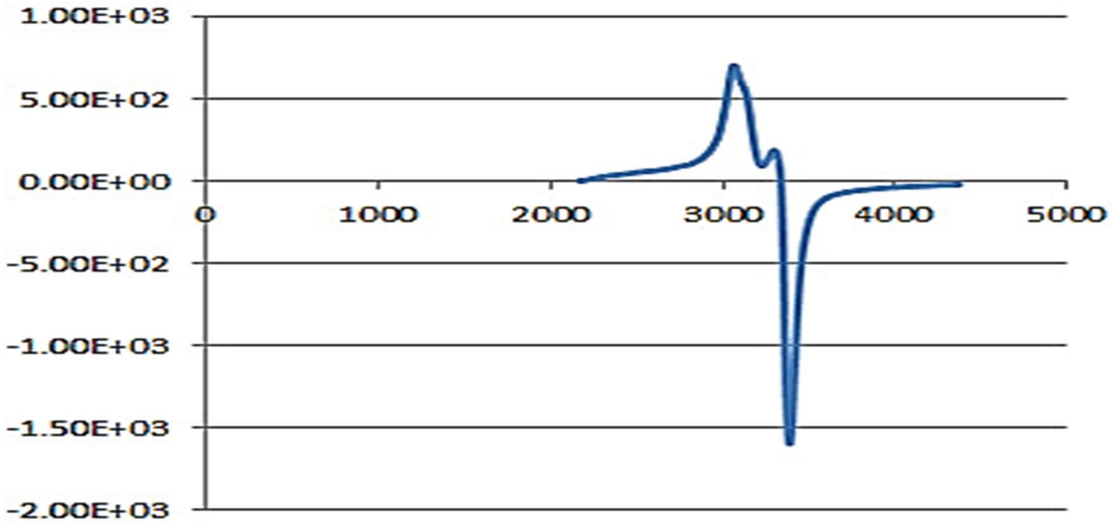

EPR spectra of complex **2**

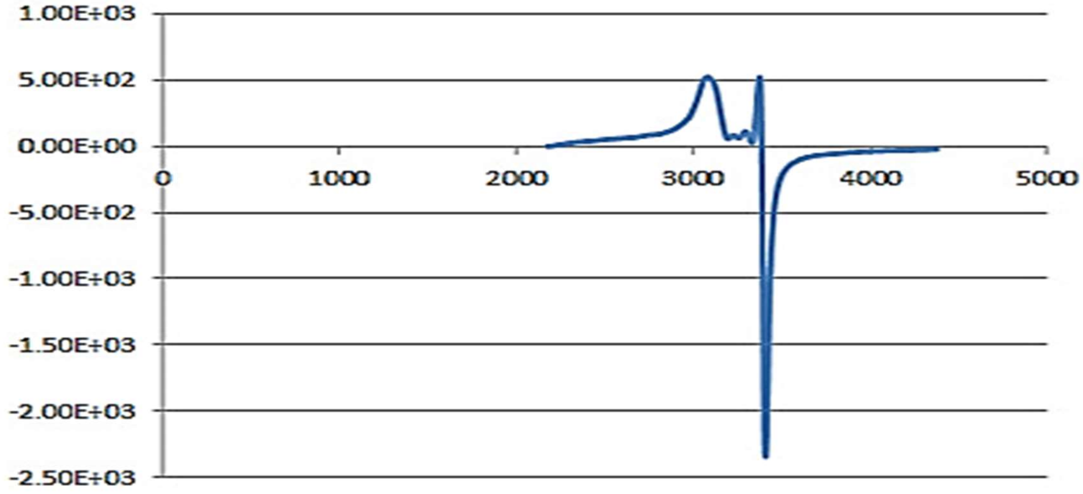

EPR spectra of complex **3**

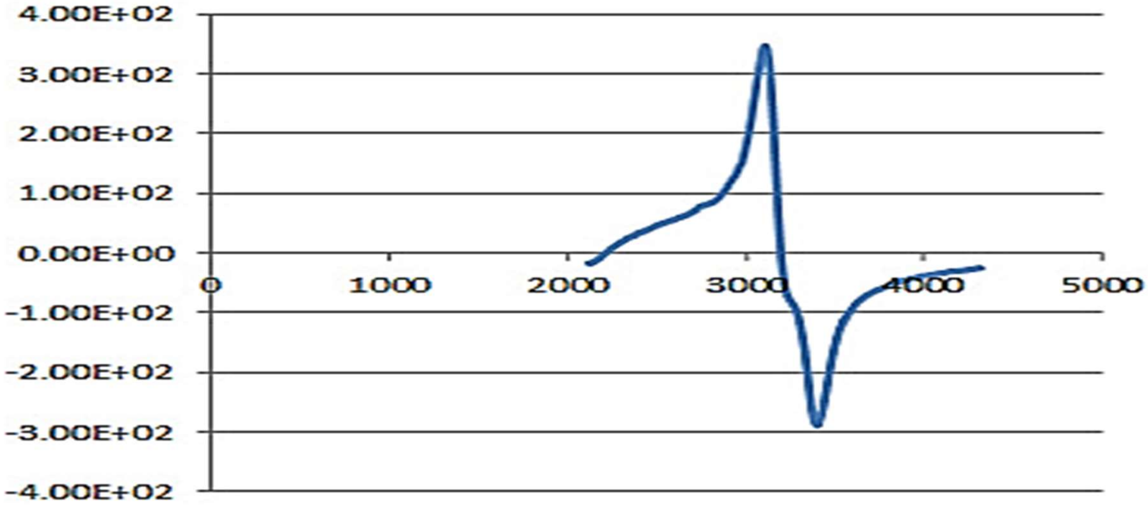

EPR spectra of complex **4**

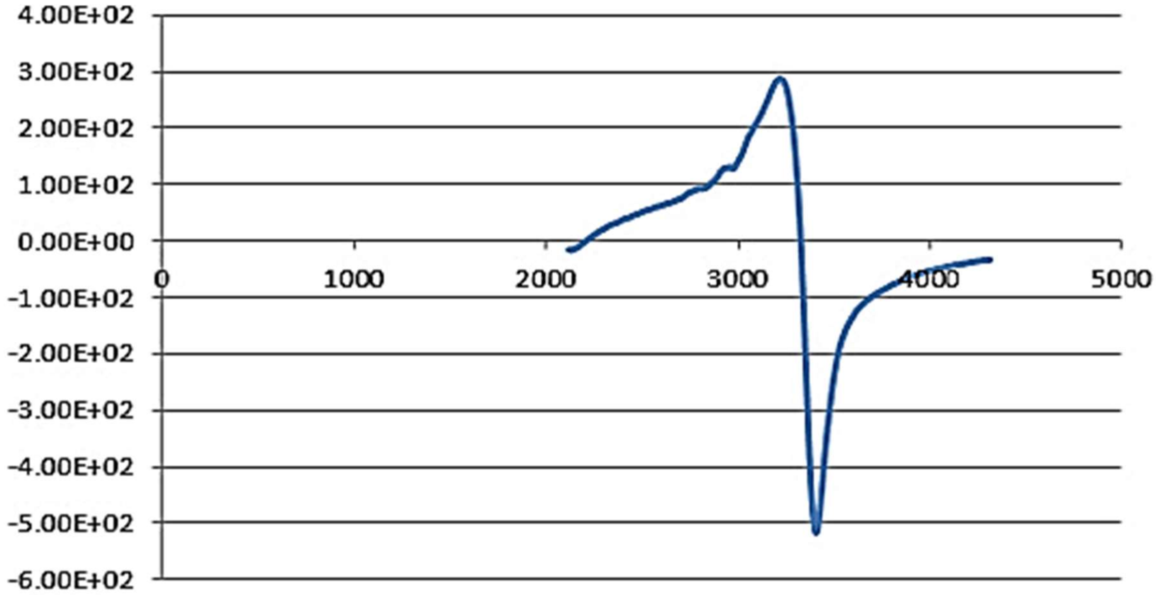

EPR spectra of complex **5**

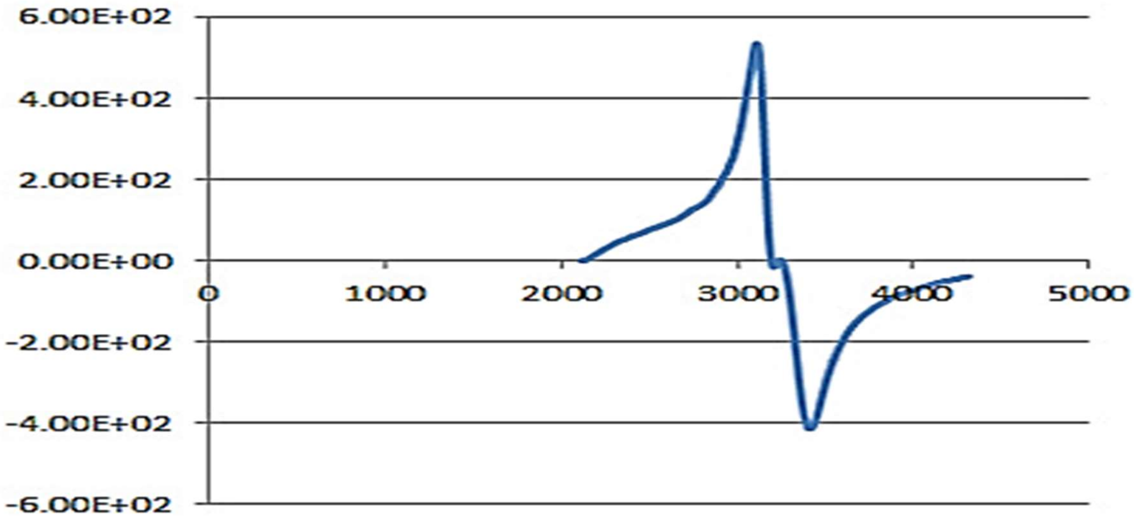

EPR spectra of complex **6**

TGA spectra

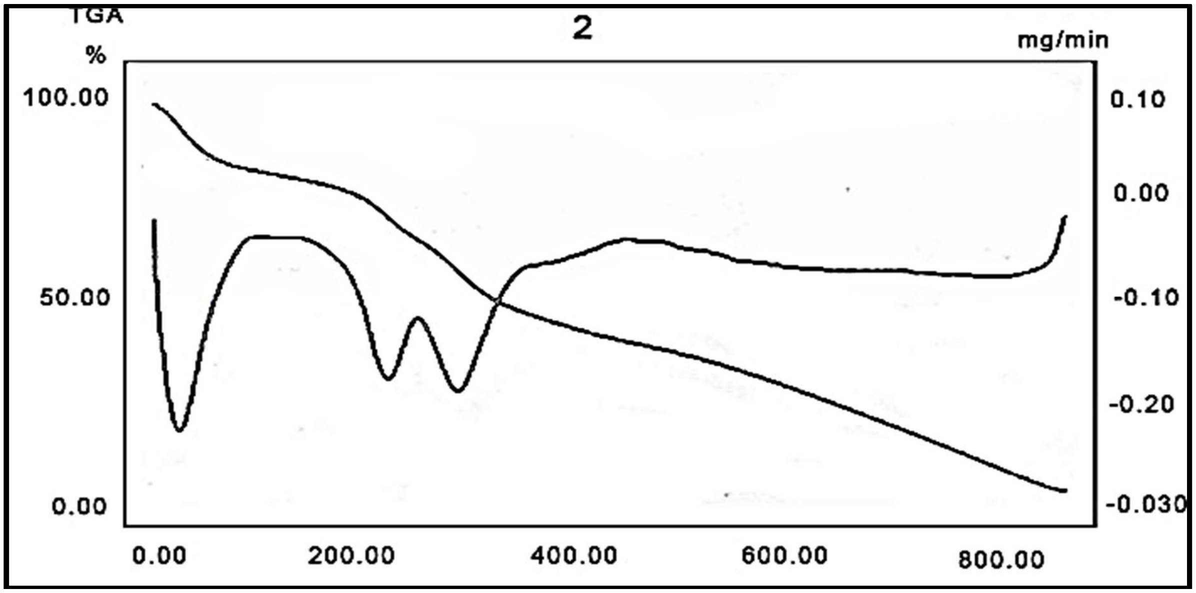

TGA spectra of complex **2**

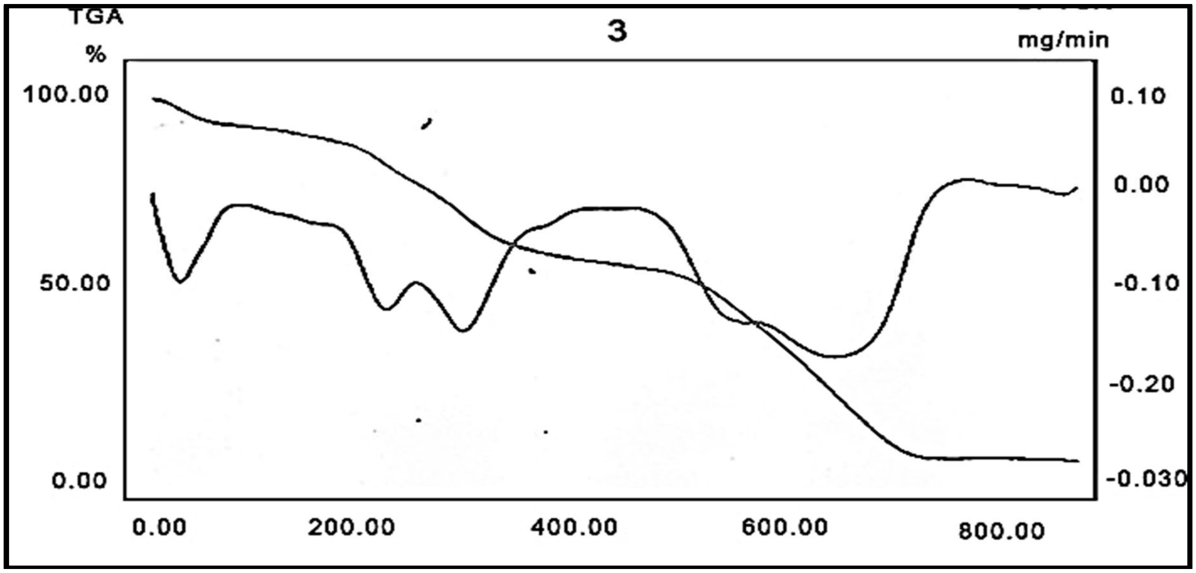

TGA spectra of complex **3**

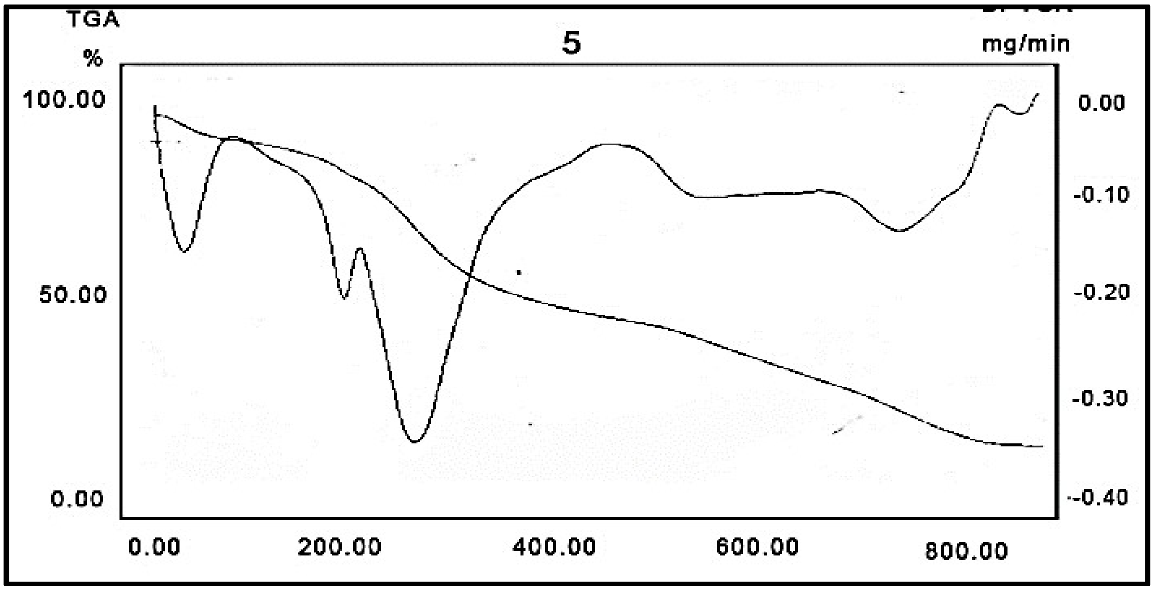

TGA spectra of complex **5**

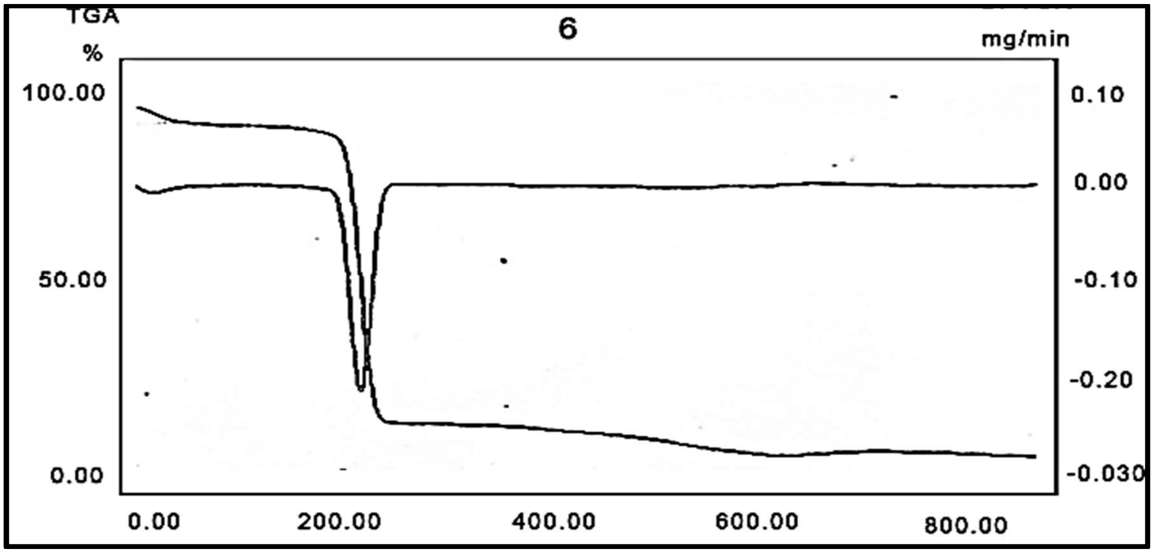

TGA spectra of complex **6**

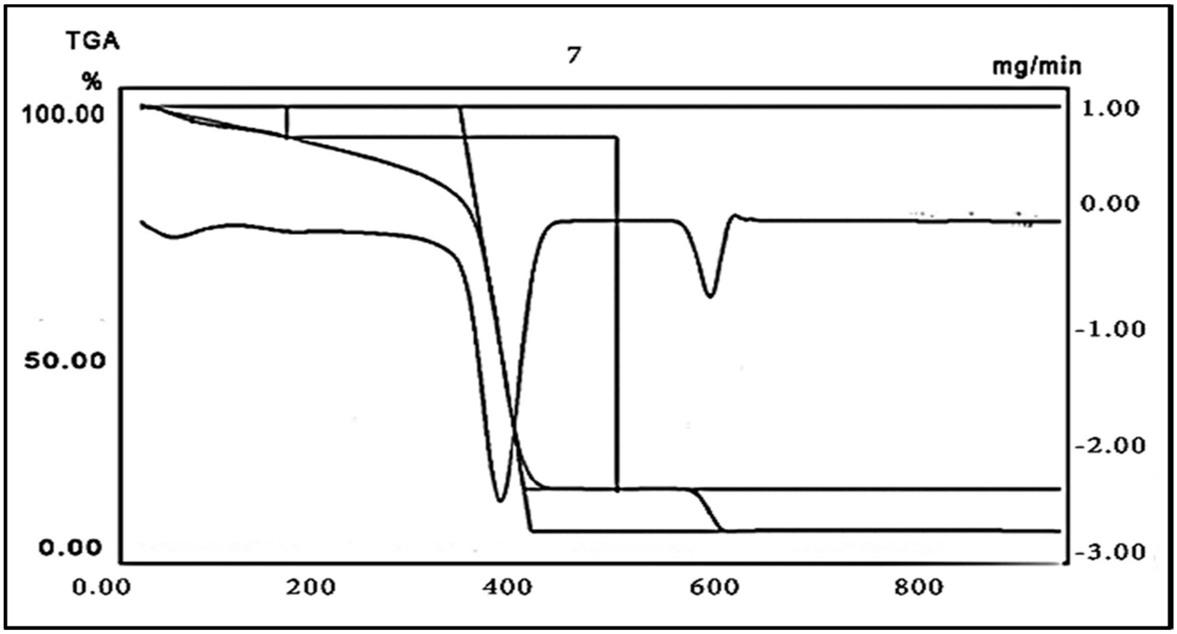

TGA spectra of complex **7**

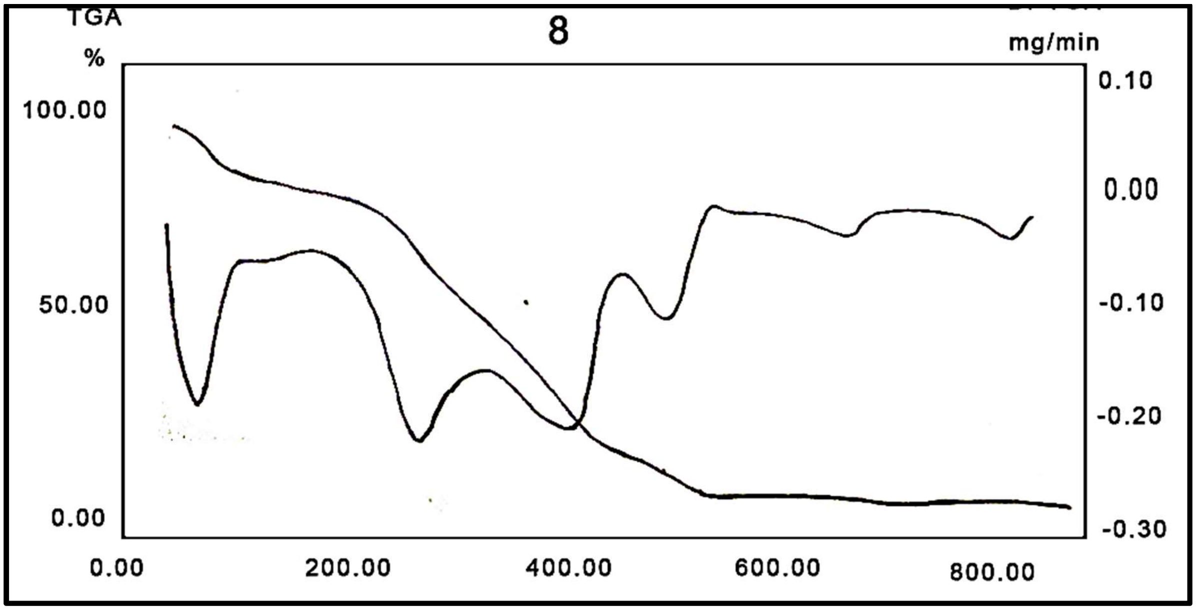

TGA spectra of complex **8**

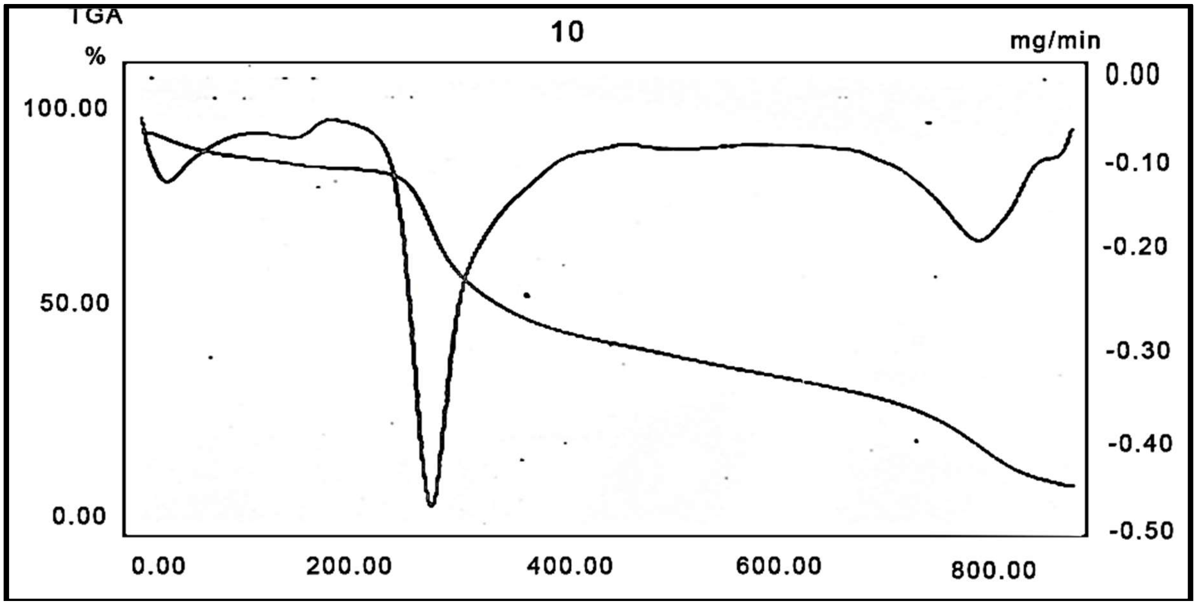

TGA spectra of complex **10**

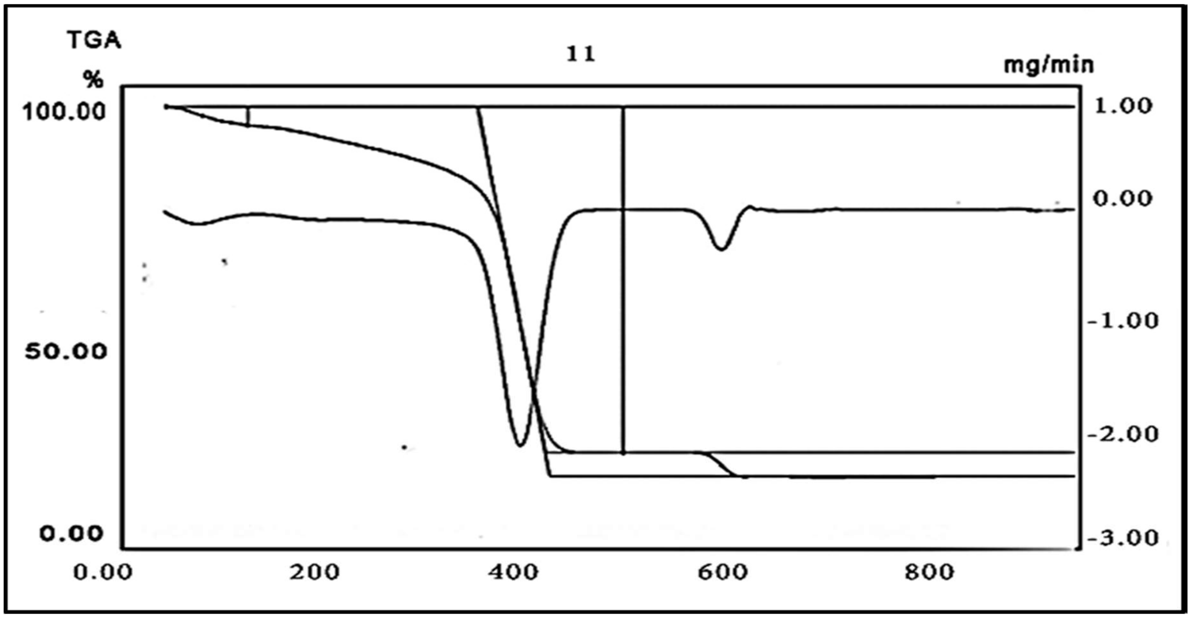

TGA spectra of complex **11**

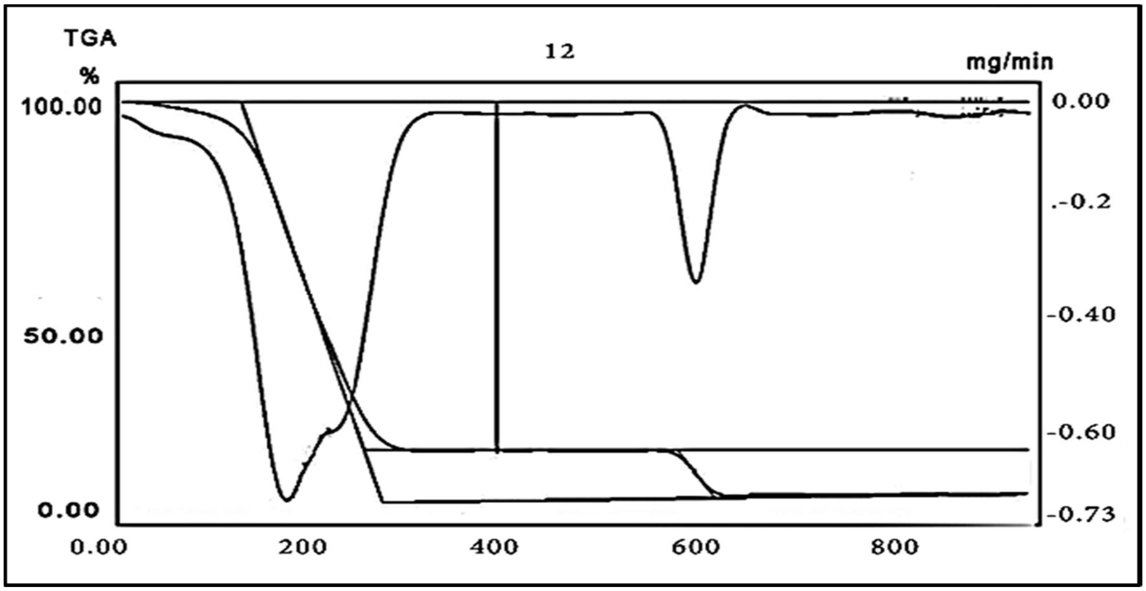

TGA spectra of complex **12**

UV-Visible spectra

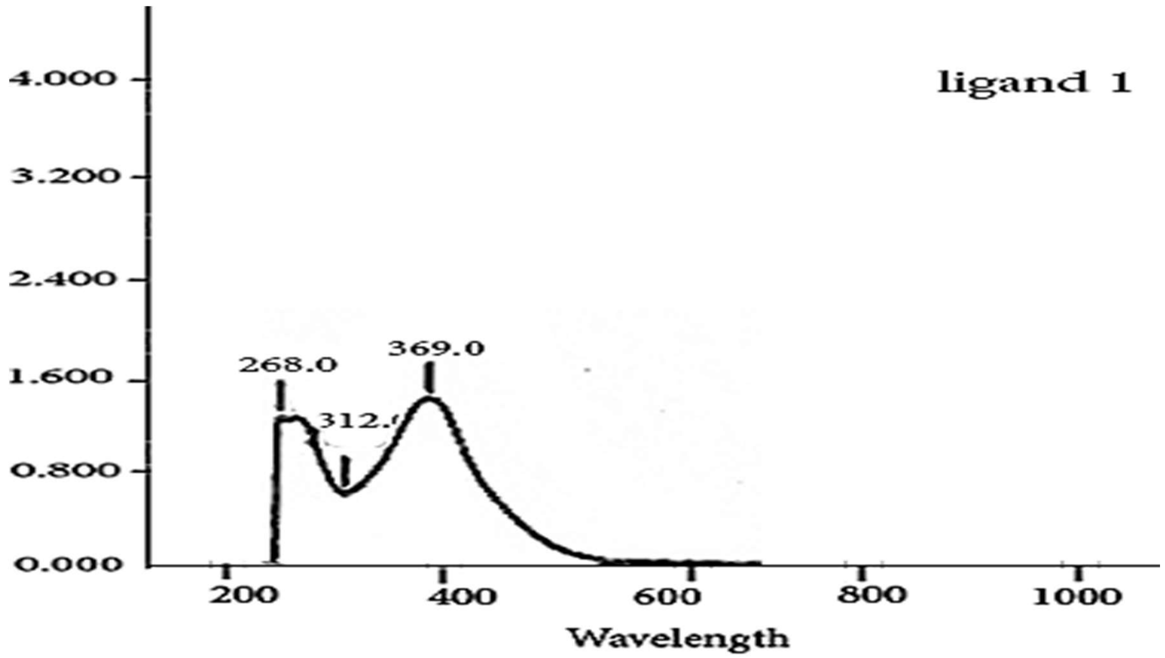

UV-Visible spectra of ligand **1**

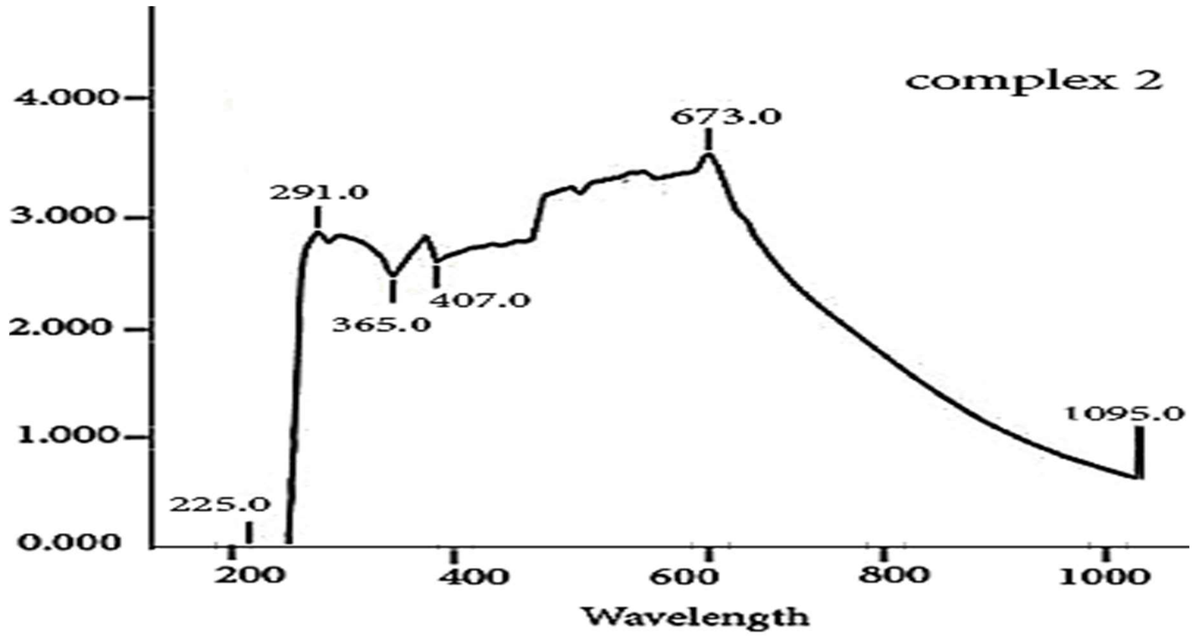

UV-Visible spectra of complex **2**

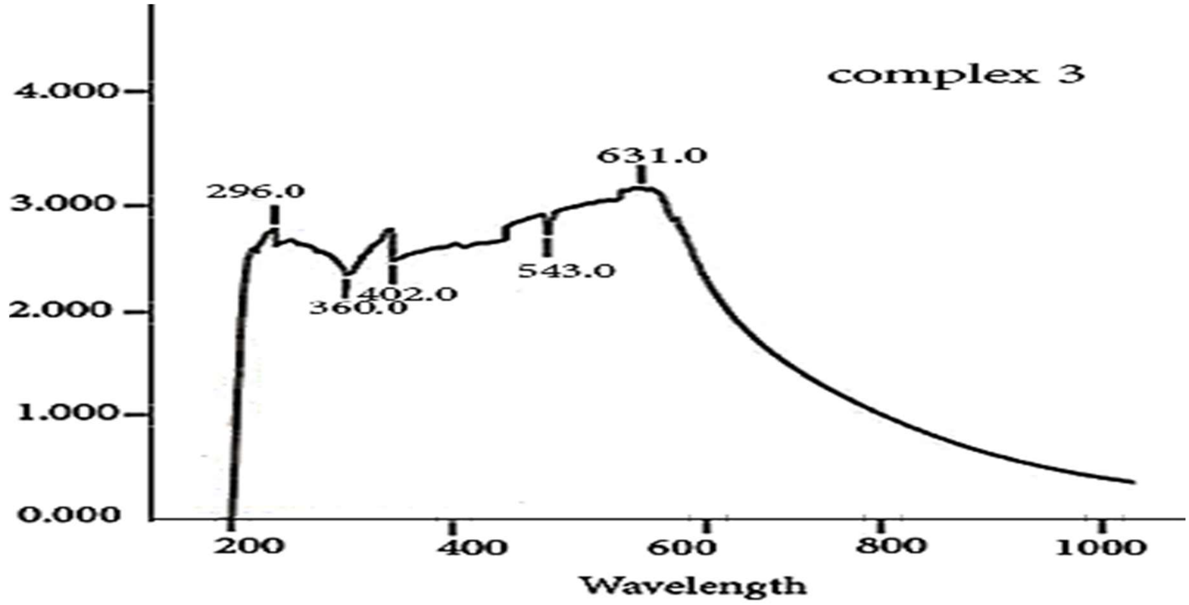

UV-Visible spectra of complex **3**

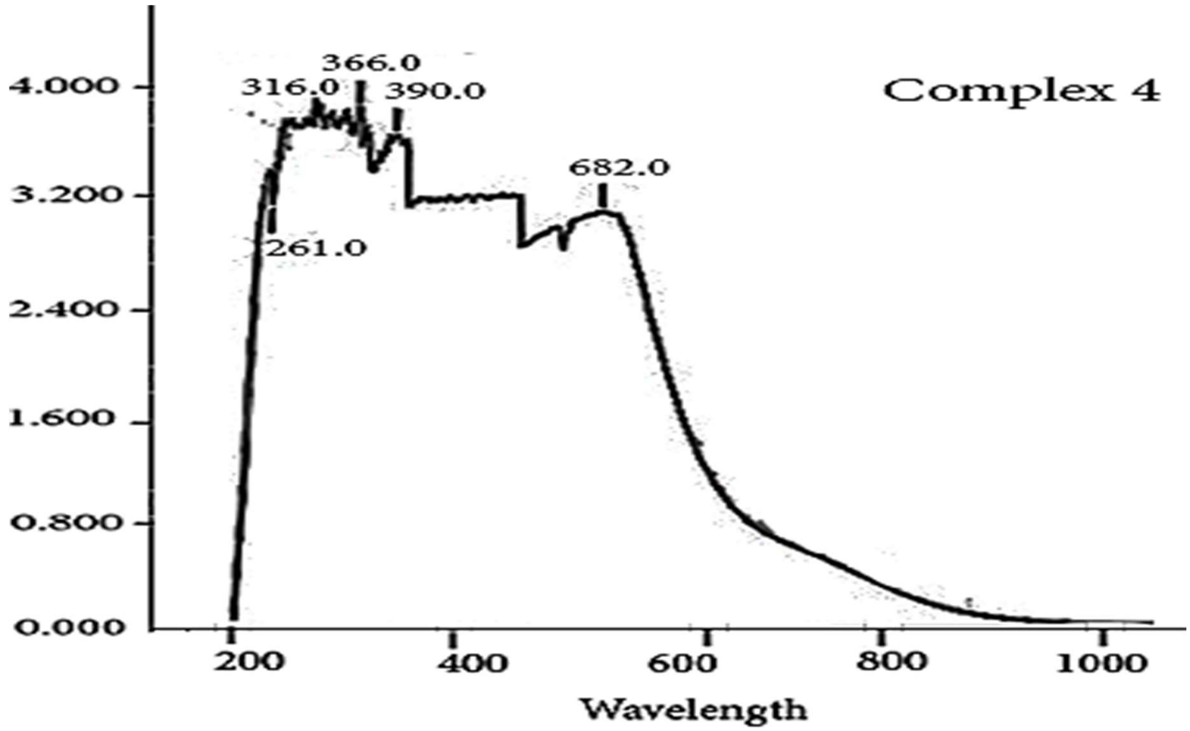

UV-Visible spectra of complex **4**

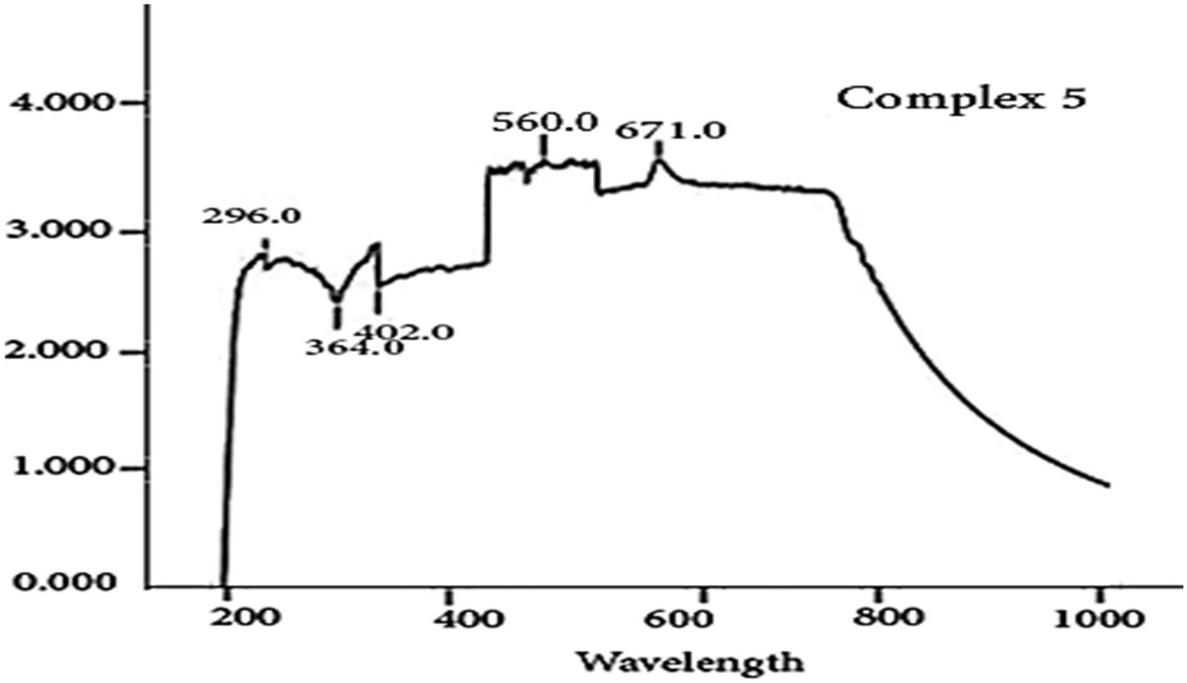

UV-Visible spectra of complex **5**

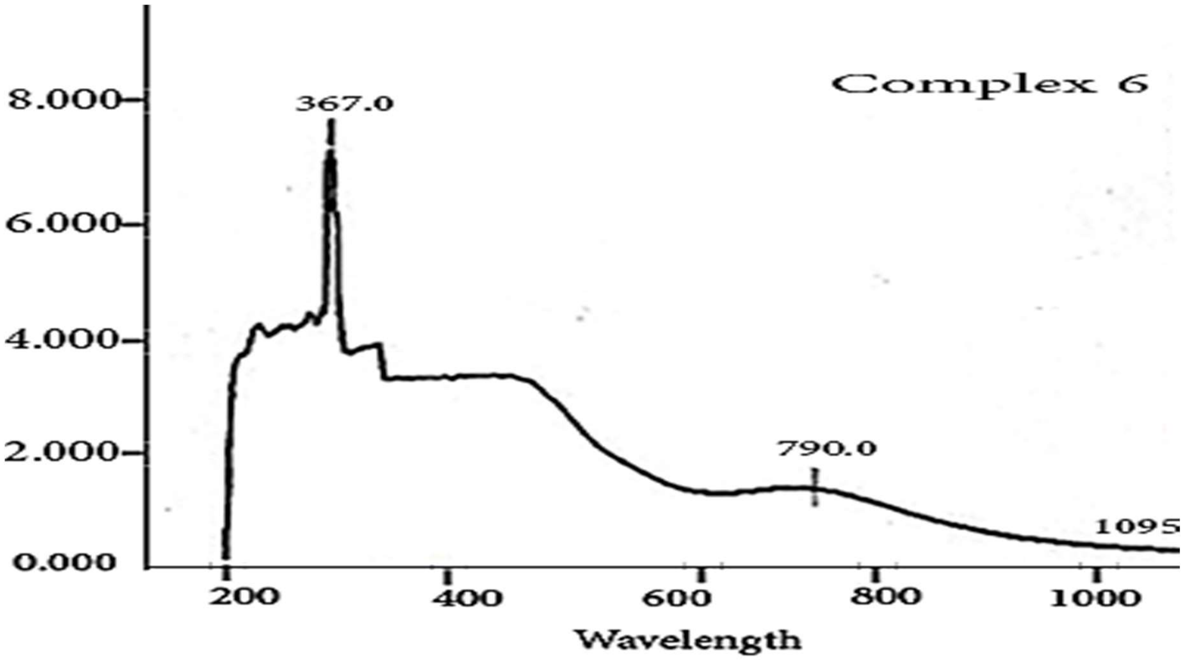

UV-Visible spectra of complex **6**

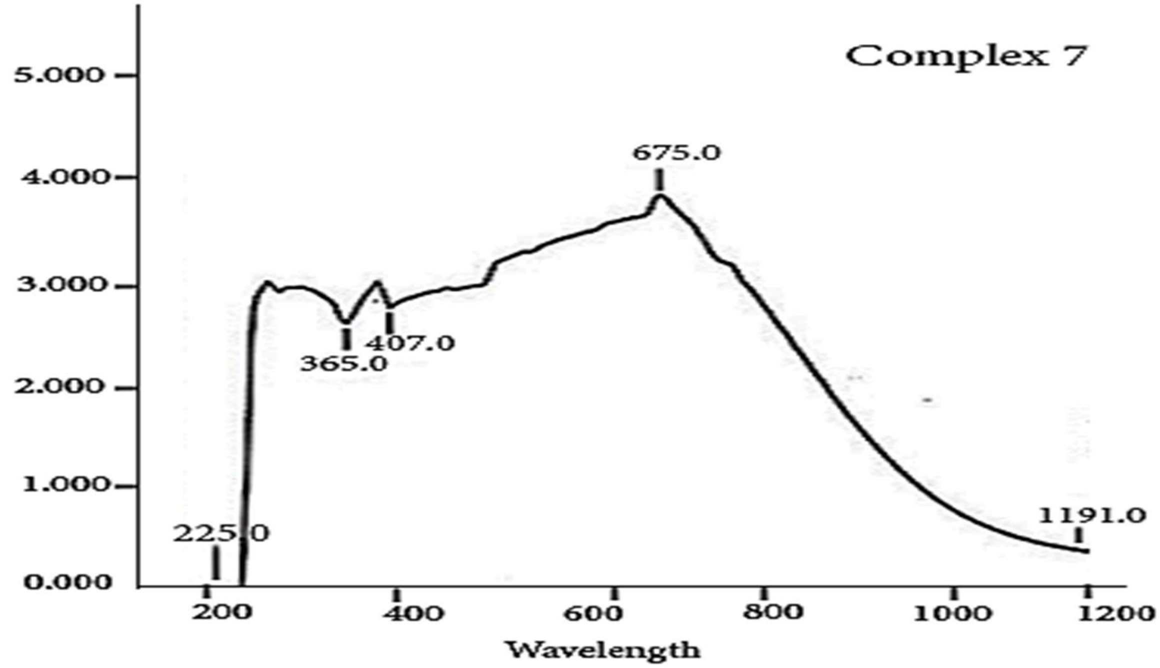

UV-Visible spectra of complex **7**

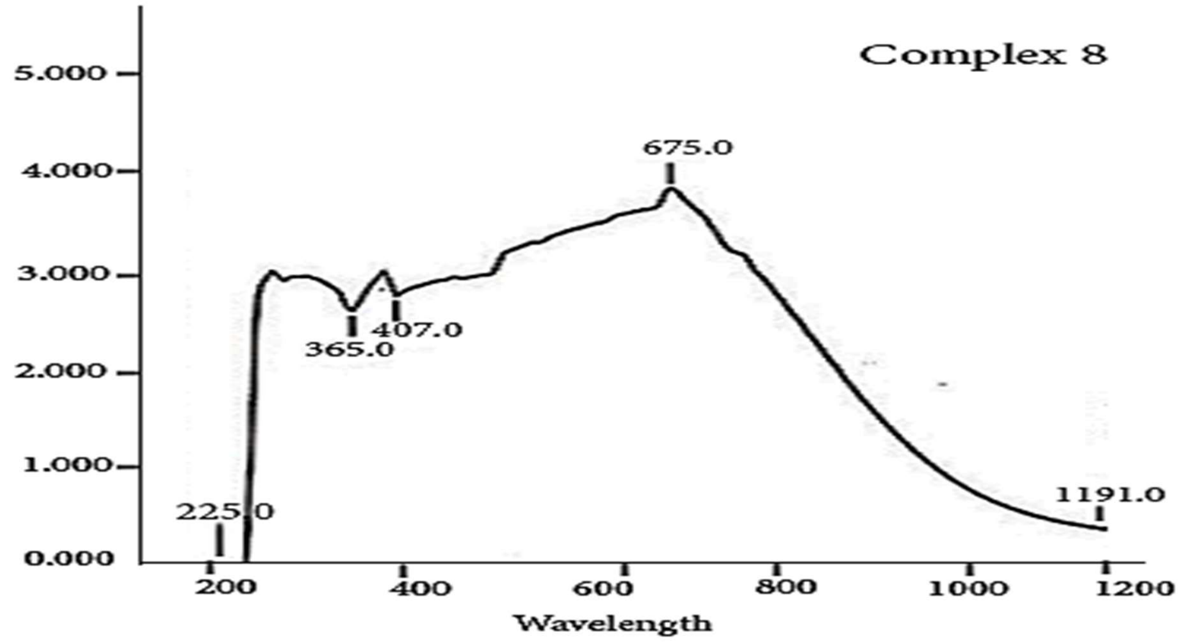

UV-Visible spectra of complex **8**

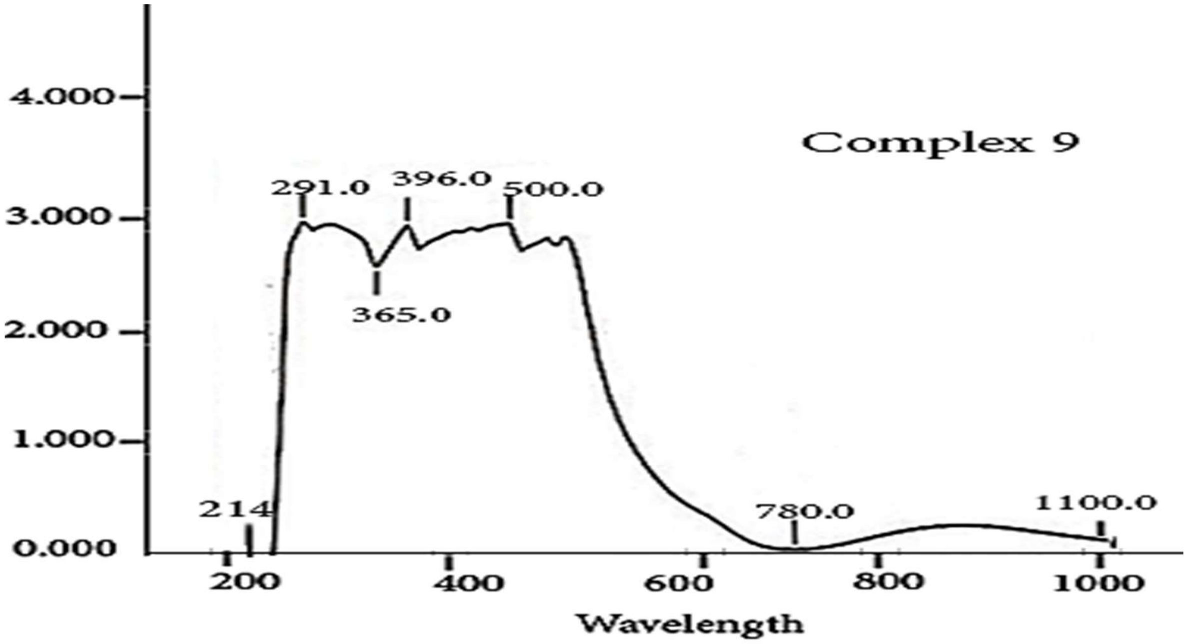

UV-Visible spectra of complex **9**

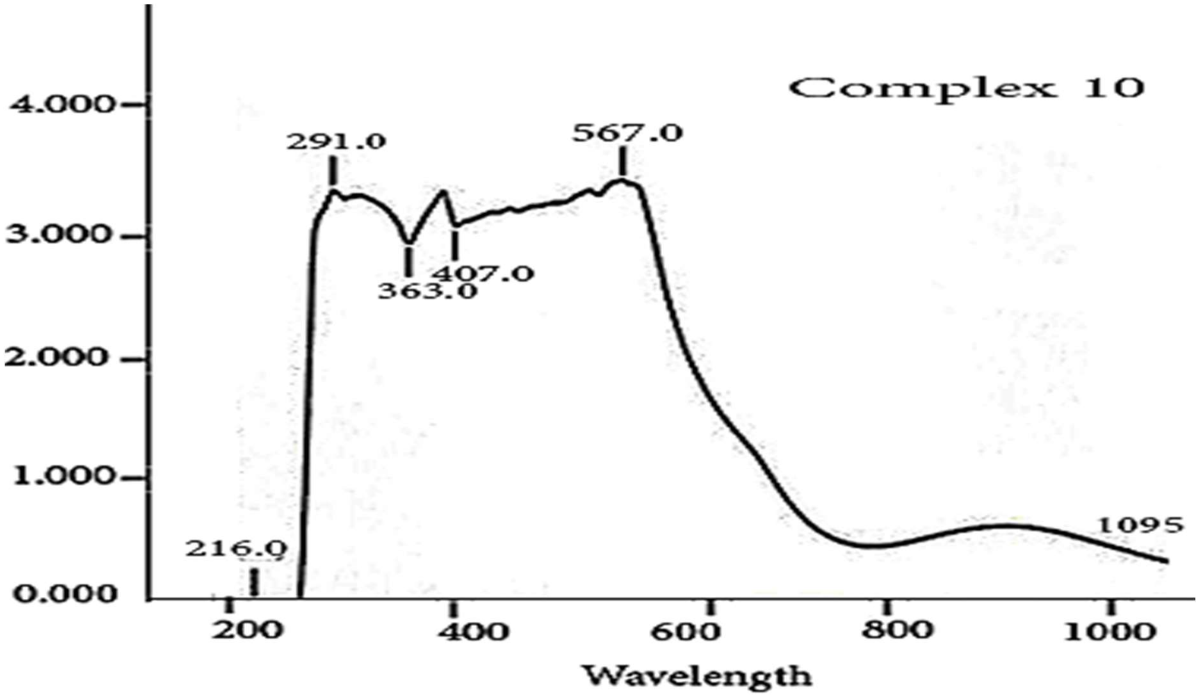

UV-Visible spectra of complex **10**

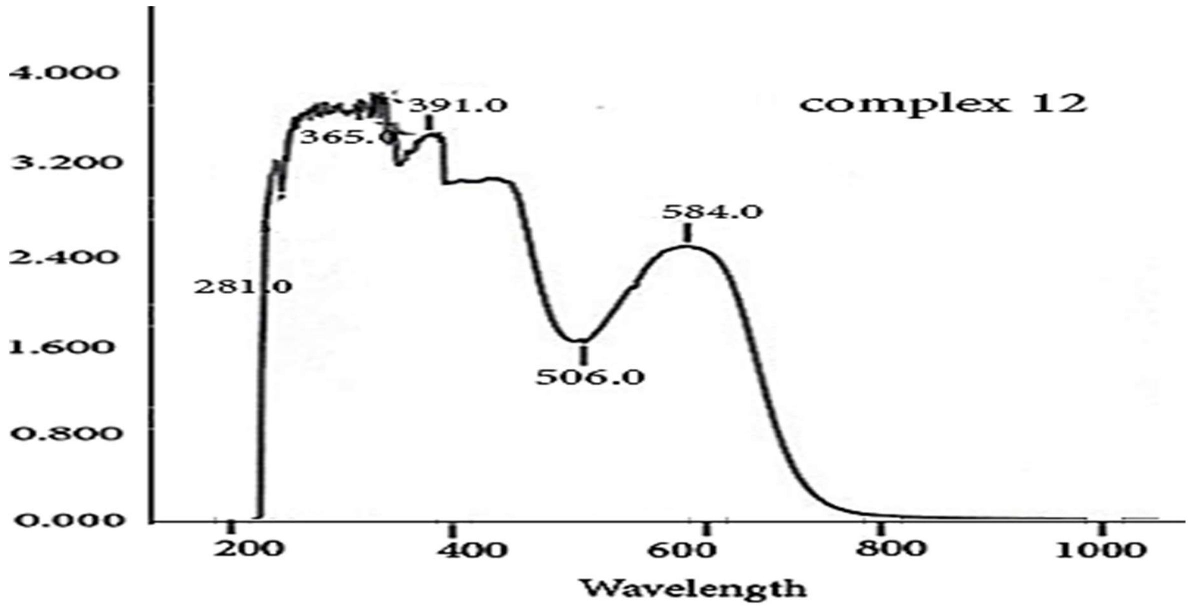

UV-Visible spectra of complex **12**
